# The Role of Metabolic Testing in the Diagnostic Evaluation of Adult NORSE: A Retrospective, Single‐Centre Study

**DOI:** 10.1111/ene.70218

**Published:** 2025-06-05

**Authors:** Jennifer Kilmer, George Ransley, Elaine Murphy, Michael G. Hanna, Robert D. S. Pitceathly, Sanjeev Rajakulendran, Chiara Pizzamiglio

**Affiliations:** ^1^ UCL Queen Square Institute of Neurology London UK; ^2^ The National Hospital for Neurology and Neurosurgery, Queen Square London UK; ^3^ Charles Dent Metabolic Unit The National Hospital for Neurology and Neurosurgery, Queen Square London UK; ^4^ Department of Neuromuscular Diseases UCL Queen Square Institute of Neurology London UK; ^5^ NHS Highly Specialised Service for Rare Mitochondrial Disorders, Queen Square Centre for Neuromuscular Diseases The National Hospital for Neurology and Neurosurgery London UK

**Keywords:** diagnosis, metabolic workup, mitochondrial diseases, NORSE, status epilepticus

## Abstract

**Background:**

New‐onset refractory status epilepticus (NORSE) is a diagnostically challenging and severe epileptic presentation in which aetiology is an important predictor of outcome. This retrospective study aimed to investigate the utility of metabolic screening to determine the underlying cause in 42 patients with suspected NORSE, admitted to The National Hospital for Neurology and Neurosurgery, London, between 2004 and 2021.

**Methods:**

Demographic, clinical, biochemical, and molecular data were collected. Sixty‐two per cent of the cohort was classified as cryptogenic (cNORSE), while 38% had symptomatic NORSE (sNORSE).

**Results:**

Despite extensive investigations (100 metabolic‐related tests were performed among the 42 cases), inherited disorders of metabolism were not identified as causes for NORSE. Nevertheless, three patients with refractory status epilepticus (RSE), who did not fulfill the diagnostic criteria for NORSE, had a primary mitochondrial disease (PMD). These data help establish criteria that distinguish PMD‐related RSE from cNORSE, including pre‐existing multisystemic features, a positive family history and/or suggestive MRI findings.

**Conclusion:**

The study highlights the challenges in diagnosing NORSE aetiology and the limited utility of extensive testing for inherited metabolic disorders in this patient population. Further research is required to refine diagnostic strategies and enhance our understanding of the heterogeneous aetiology of cNORSE.

## 1 Introduction

New‐onset refractory status epilepticus (NORSE) is defined as prolonged seizure activity refractory to first and second‐line antiseizure medications (ASMs), with no obvious aetiology determined in the first 72 h, without a prior history of epilepsy [1]. NORSE has a high mortality rate (16%–27%), and poor functional outcomes in up to two‐thirds of survivors [2].

A key determinant of outcome in status epilepticus is the underlying cause, which underscores one of the principal challenges of managing NORSE, where the cause remains unknown in up to 50% [3]. Such ‘cryptogenic’ NORSE (cNORSE) cases potentially run a more severe course [2].

While inherited metabolic disorders (IMDs), particularly primary mitochondrial diseases (PMDs), are considered in the diagnostic work‐up of NORSE typically when autoimmune and infectious causes are excluded, their role in adult‐onset NORSE is unclear and their prevalence has not been systematically studied [4, 5]. This often entails extensive genetic and biochemical tests in blood, CSF, and muscle.

This study aims to address this gap by evaluating the utility of metabolic screening in a cohort of adult‐onset NORSE to enhance the diagnostic process and inform clinical decision making.

## 2 Methods

This retrospective study reviewed all cases aged 16 years or older admitted to The National Hospital for Neurology and Neurosurgery (NHNN) neurological intensive care unit (ICU) with refractory SE (RSE) between January/2004 and December/2021. Of these, we have subsequently identified cases with NORSE as defined by Hirsch et al. [1] Cases were further categorised into symptomatic NORSE (sNORSE) or cNORSE based on the presence or absence of a final diagnosis. Twenty‐five NORSE cases were previously reported elsewhere [6].

Patient data, including demographics, clinical variables, outcomes and test results, were extracted from hospital records. Outcomes at discharge and 1‐year follow‐up were rated using the modified Rankin Scale (mRS) for neurological disability [7]. Mortality rates were ascertained at three timepoints: during admission, at 1‐year follow‐up, and through to July/2022 (end of observation). Results of a comprehensive battery of second‐line IMD investigations were designated normal or abnormal based on standard reference ranges available from the NHNN laboratory. The clinical relevance of these tests was established based on clinician opinions documented in the final report. The decision to conduct second‐line IMD investigations, including the type of investigations, was made by the clinician on a case‐by‐case basis.

Results throughout this study are presented as median (interquartile range [IQR]) or mean ± standard deviation (SD). Data were compared using Mann Whitney *U* test or Pearson's chi‐squared test. A *p* value < 0.05 was considered significant. Analyses were performed with SPSS (v. 19.0 for Windows; SPSS Inc.).

This study was submitted to the Clinical Audit and Quality Improvement Subcommittee at University College London Hospitals Trust. Informed consent was not required as the data were collected as part of routine clinical practice.

## 3 Results

### 3.1 Demographics and Clinical Characteristics

Demographics and clinical characteristics are summarised in Tables S1–S4, Figure S2. A total of 89 patients with SE were admitted to the NHNN ICU between January/2004 and December/2021. Of these, 73 were classified as RSE and 42 (57.5%) met the criteria for NORSE (Figure S1). An aetiology was determined in 16 cases (38%), while the remaining 26 cases (62%) were classified as cNORSE.

In the NORSE cohort there were more females (59.5%) and the median age was 28 years. The median ICU stay was 72 days, and the hospital stay was 105 days. Outcomes were generally poor, with 83.4% of patients having an mRS score ≥ 4 at discharge, indicating moderately severe disability or worse. The in‐hospital mortality rate was 15.4%, which increased to 20.5% at 1 year. There were no significant differences between cNORSE and sNORSE cases in terms of mortality rates and mRS outcomes.

### 3.2 Metabolic Investigations

IMD investigations were performed in 50% of patients (*n* = 21). Of these, PMD was the most common disorder (10/21). These tests were more commonly performed in cNORSE compared to sNORSE (*p* < 0.001 for IMD, and *p* = 0.04 for PMD), Figure S4, Table S5.

Table 1 outlines the type and frequency of metabolic investigations completed in our NORSE cohort, grouped as defined by Almannai et al. [8] Most metabolic test results were normal, and all cases of abnormal results were deemed clinically irrelevant. Despite constituting a considerable proportion of the average work‐up (100 IMD second‐line investigations), none of these tests contributed to identifying the NORSE aetiology in our cohort. Muscle histopathology and respiratory chain enzyme (RCE) analyses did not reveal any features of metabolic pathology, including mitochondrial dysfunction.

**TABLE 1:** Metabolic and other genetic disorders investigated in the NORSE cohort. Most metabolic test results were normal, and all cases of abnormal results were deemed clinically irrelevant. The categorisation of metabolic disorders is based on those defined in Almannai et al. [8] The routine standard metabolic tests are reported here for completion but only the IMD‐second line investigations are discussed in the paper.

Test name	Completed	Abnormal
	*N*°	*N*° (%)
**IMD second‐line investigations (total *N*° = 100)**		
Aminoacidopathies/Organic acidemias/Urea cycle defects (*N*° = 36)		
Urine/Plasma amino acid profile	19	1 (5.3)^i^
Urine organic acids	9	3 (33.3)^i^
Acyl carnitine profile^a^	6	0 (0)
Methylmalonic acid	2	0 (0)
Disorders of energy metabolism (*N*° = 35)		
Mitochondrial genetic testing		
mtDNA common point mutations^b^	10	0 (0)
Maintenance panel^c^	5	0 (0)
Whole mtDNA sequencing	2	0 (0)
Whole exome sequencing (WES)	2	0 (0)
Muscle biopsy^d^	6	1 (16.7)^k^
Urine/plasma creatine and guanidinoacetate	4	0 (0)
Respiratory chain enzyme analysis (RCEA) in muscle	3	0 (0)
FGF21	3	0 (0)
Cofactor‐related disorders (*N*° = 14)		
CSF pterins^e^	5	0 (0)
CSF serotonergic/dopaminergic metabolites^f^	4	0 (0)
Biotinidase	3	0 (0)
ɑ‐AASA	1	0 (0)
*FOLR1* genetic testing	1	0 (0)
Purine/Pyrimidine disorders (*N*° = 5)		
Urine purines/pyrimidines	4	0 (0)
Lesch–Nyhan genetic testing (*HPRT*)	1	0 (0)
Peroxisomal/lysosomal disorders (*N*° = 10)		
White cell enzyme activity	5	0 (0)
Very long chain fatty acid profile^g^	4	0 (0)
Bile acid intermediates	1	0 (0)
**Routine** **standard** **metabolic** **tests** **(** **total** *N* **=** **1**1**3** **)**		
Ammonia	25	16 (64)
Glucose	24	6 (25)
CSF lactate	20	2 (10)
Serum lactate	19	3 (15.8)
Serum folate	15	4 (26.7)
Homocysteine	10	3 (30)^h^

Abbreviations: ɑ‐AASA = alpha‐aminoadipic semialdehyde dehydrogenase, FGF21 = fibroblast growth factor 21, FOLR1 = folate receptor alpha gene, HPRT = hypoxanthine‐guanine phosphoribosyltransferase.

^a^ Acylcarnitines, free carnitine, total carnitine.

^b^ mtDNA common point mutations include m.3243A>G in *MT‐TL1*, m.8344A>G in *MT‐TK*, and m.8993T>G/C in *MT‐ATP6*.

^c^ Panel of nuclear genes involved in mtDNA maintenance includes: *ABAT*, *AFG3L2*, *DGUOK*, *DNA2*, *DNM2*, *FBXL4*, *MFN2*, *MGME1*, *MPV17*, *OPA1*, *POLG*, *POLG2*, *RNASEH1*, *RRM2B*, *SLC25A4*, *SPG7*, *SUCLA2*, *SUCLG1*, *TFAM, TK2*, *TOP3A*, *TWNK*, *TYMP*.

^d^ Analysis performed on muscle tissue included large‐scale mtDNA rearrangements (*n* = 3) and mtDNA depletion (*n* = 1).

^e^ Dihydrobiopterin, neopterin, methyltetrahydrofolate, pyridoxal phosphate.

^f^ Homovanilic acid, 5‐hydroxyindoleactic acid, HVA:5HIAA.

^g^ C22, C24, C26, C24:C22 ratio, C26:C22 ratio, phytanic acid, pristanic acid.

^h^ Secondary to B12/folate deficiency.

^i^ Clinically irrelevant.

^k^ Findings consistent with disuse atrophy.

Of the 100 IMD investigations, 21 were genetic tests performed in 10 patients (24%). PMD testing constituted the vast majority, accounting for 19/21 genetic tests performed in 10 patients (nine with cNORSE and one with a final diagnosis of autoimmune sNORSE). All tests were negative. Tests included: common mtDNA pathogenic point mutations (m.3243A>G, m.8344A>G, and m.8993T>G/C) (*n* = 10), panel‐based testing of 23 relevant mitochondrial nuclear‐encoded genes including *POLG* (*n* = 5), whole sequencing of the mitochondrial genome (*n* = 2), and whole exome sequencing (*n* = 2). Metabolic, non‐mitochondrial genetics included analysis of *HPRT* (Lesch–Nyhan syndrome) (*n* = 1) and *FOLR1* (cerebral folate transport deficiency) (*n* = 1).

Additional non‐metabolic genetic tests (mostly linked with epileptic encephalopathies) were performed in eight patients, all with negative results (Table S5).

### 3.3 Inherited Metabolic Disorders in RSE

While no IMD was identified in the NORSE cohort, three PMD cases (out of seven tested) were found in the RSE group (Figure S1), none meeting NORSE criteria. One case had the m.3243A>G, *MT‐TL1* pathogenic variant in mtDNA (heteroplasmy: 17% in blood, 89% in urine). The phenotype was consistent with myoclonic epilepsy with ragged red fibres (MERRF)/Mitochondrial encephalomyopathy, lactic acidosis and stroke‐like episodes (MELAS) overlap. The second one had autosomal recessive pathogenic variants in *POLG* (c.1399G>A, p.Ala467Thr). A third patient showed multiple mtDNA deletions in muscle, suggesting a nuclear gene defect of mtDNA maintenance, but no genetic confirmation was detected. Interestingly, differences between the PMD‐related RSE and the 10 NORSE cases where PMD was ruled out through testing can be noted.

Firstly, the PMD‐related RSE had a previous history of epilepsy, indicating that the initial presentation of PMD with NORSE is uncommon. PMD imaging (Figure S3) revealed pronounced bilateral cerebellar atrophy at the SE onset‐MRI, thus suggesting an ongoing chronic process besides the acute SE (Figure S3b). Notably, metabolic infarcts were observed in two cases (m.3243A>G and *POLG* pathogenic variant) (Figure S3a,c). These were in contrast with the normal or nonspecific changes observed in the cNORSE cases (Figure S3e), or with the infectious‐related sNORSE (Figure S3d).

Similarly, the muscle biopsy of the *POLG*‐related PMD demonstrated abnormalities consistent with mitochondrial alterations, including ragged red fibres with reduced or absent COX activity, while none of the six biopsies in the cNORSE cases exhibited such features.

## 4 Discussion

This study evaluated 42 NORSE patients, the youngest aged 18, with 26 (62%) classified as cryptogenic. Investigations conducted after excluding infectious and autoimmune causes did not identify any metabolic diagnosis in our cohort.

IMD, a rare and diverse group of genetic disorders affecting metabolic pathways, is reported as a ‘rare’ cause of SE, and a list of IMD‐associated genes, mostly including mitochondrial genes, has been described [4]. Specific cases of NORSE involve mtDNA mutations (e.g., *MT‐TF1*) and nDNA mutations (e.g., *POLG*, *DNM1L*, *FASTKD2*), though most reports involve single cases, primarily in children [9, 10, 11, 12]. Among these IMDs, some potentially treatable causes of NORSE, such as CAD deficiency, should not be overlooked [13].

PMD‐related RSE cases differed from the cNORSE cohort who underwent mitochondrial genetic testing in age, clinical history and outcomes. PMD patients, in whom there is often a previous history of severe epilepsy and multisystemic involvement, showed distinct radiological features, such as chronic abnormalities, metabolic strokes and mitochondrial dysfunction on muscle biopsy. In contrast, cNORSE patients were younger (28.3 vs. 37.7 years), neurologically healthy, and lacked these features. The prognosis for PMD‐related RSE was worse, with all PMD cases deceased compared to 2/10 NORSE patients. Given these findings, the likelihood of an undiagnosed IMD in previously healthy adults presenting with NORSE is extremely low, especially with normal imaging.

**FIGURE 1:**
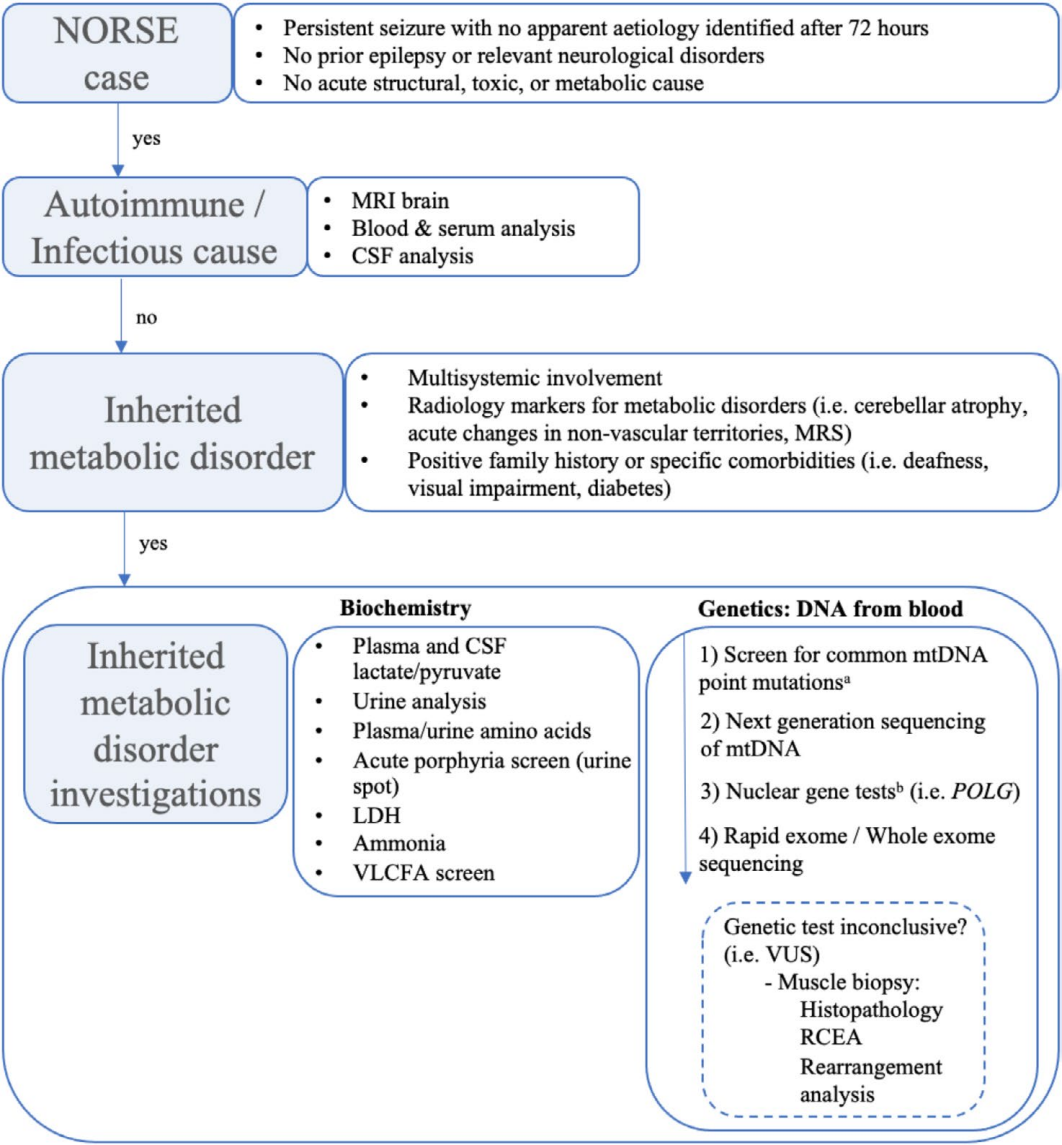
Suggested flowchart of metabolic investigations in NORSE. ^a^It includes m.3243A>G in *MT‐TL1*, m.8344A>G in *MT‐TK*, and m.8993T>G/C in *MT‐ATP6*. ^b^Includes *ABAT*, *AFG3L2*, *DGUOK*, *DNA2*, *DNM2*, *FBXL4*, *MFN2*, *MGME1*, *MPV17*, *OPA1*, *POLG*, *POLG2*, *RNASEH1*, *RRM2B*, *SLC25A4*, *SPG7*, *SUCLA2, SUCLG1*, *TFAM*, *TK2*, *TOP3A*, *TWNK*, *TYMP*. LDH = lactate dehydrogenase, MRS = magnetic resonance spectroscopy, POLG = polymerase subunit gamma, RCEA = respiratory chain enzyme analysis, VLCFA = very long chain fatty acids, VUS = variant of unknown significance.

PMD genetic tests in our tertiary mitochondrial centre follow a stepwise testing strategy, beginning with common mtDNA mutations, mitochondrial nuclear‐encoded gene panels and advancing to next generation sequencing (NGS) if necessary [14]. Eventually, in the ICU, the rapid exome could be adopted, to rapidly identify potentially treatable metabolic conditions [15]. Muscle tissue analysis should be reserved for cases needing verification of genetic findings, considering advancements in NGS and the limited sensitivity of traditional histopathology [14]. A proposed flowchart of investigations in cNORSE is provided in Figure 1.

This study has limitations, including the potential for sampling bias due to the specialised nature of our ICU, retrospective design, small sample size and advancements in genetic testing over the study period. These issues highlight the need for collaborative research across specialist centers to advance diagnostic understanding in NORSE.

In conclusion, while identifying the cause of NORSE remains a significant challenge, our data suggest that IMD may be less likely in the absence of other suggestive clinical, radiological features, and/or positive family history. Therefore, exploring underlying metabolic diatheses should be clinically indicated, targeted and based on a clear rationale to avoid delays in diagnosis and appropriate management.

## Author Contributions

**Jennifer Kilmer:** writing – original draft, methodology, visualization, formal analysis, data curation. **George Ransley:** methodology, investigation, writing – review and editing. **Elaine Murphy:** conceptualization, writing – review and editing, methodology. **Michael G. Hanna:** conceptualization, methodology, writing – review and editing, supervision. **Robert D. S. Pitceathly:** conceptualization, writing – review and editing, methodology, supervision, data curation. **Sanjeev Rajakulendran:** conceptualization, investigation, writing – review and editing, methodology, validation, supervision. **Chiara Pizzamiglio:** conceptualization, investigation, writing – review and editing, methodology, validation, data curation, supervision.

## Ethics Statement

All procedures followed were in accordance with the ethical standards of the responsible committee on human experimentation (institutional and national) and with the Helsinki Declaration of 1975, as revised in 2000. As the study includes only previously collected and available, non‐identifiable information, the UK Health Research Authority (HRA) was consulted and advised that it did not require review by an NHS Research Ethics Committee (REC).

## Conflicts of Interest

The authors declare no conflicts of interest.

## Supporting information


Data S1.

## Data Availability

The data that support the findings of this study are available from the corresponding author, upon reasonable request.
