# Supplementary material for: The Role of Metabolic Testing in the Diagnostic Evaluation of Adult NORSE: A Retrospective, Single‐Centre Study
Source: Eur J Neurol. 2025 Jun 5;32(6):e70218. doi: 10.1111/ene.70218 (PMC12138435; doi:10.1111/ene.70218)
Supplement: Supplementary file 1 — Data S1. [file ENE-32-e70218-s001.zip › 3_NORSE_SUPPL_File_23.05.25.docx]

**SUPPLEMENTARY FIGURE 1**


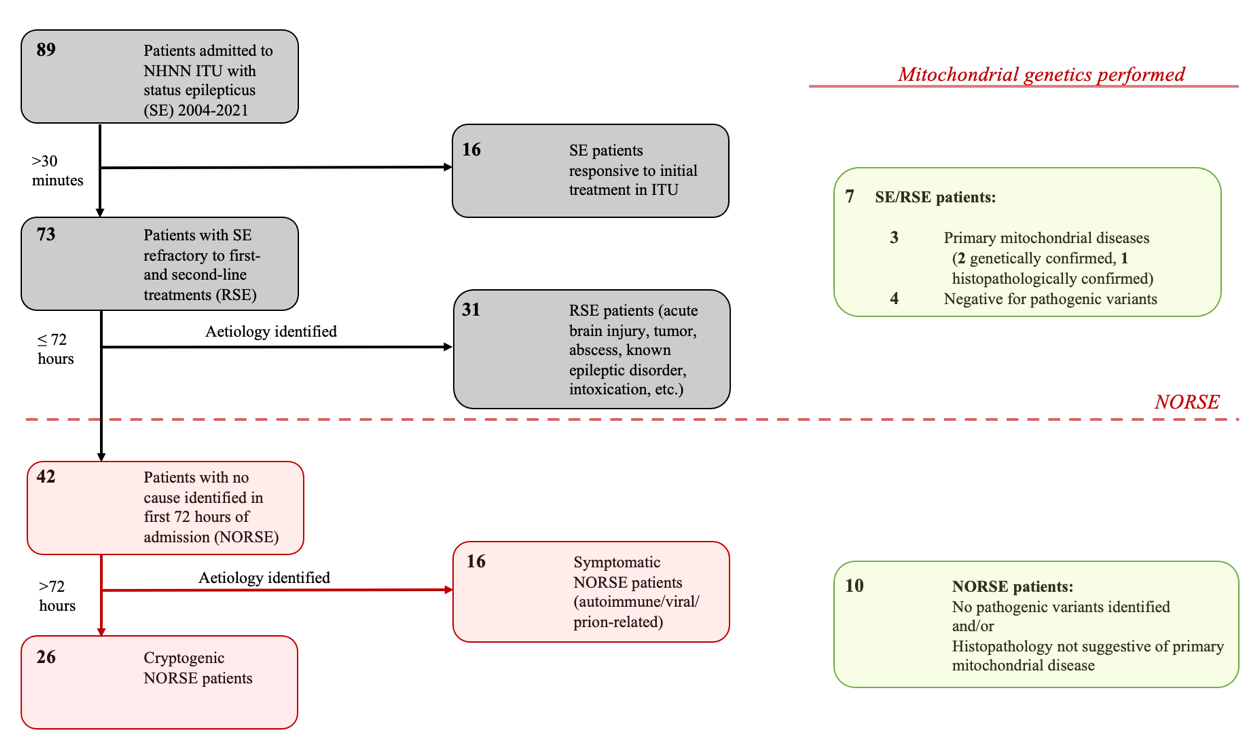


**Supplementary Figure 1:** Flowchart demonstrating the classification of the 89 patients admitted to NHNN intensive care unit with status epilepticus (SE) from 2004 to 2021, with information on mitochondrial genetic testing included.

*Abbreviations: NHNN = The National Hospital of Neurology and Neurosurgery; ITU = intensive therapy unit; RSE = refractory status epilepticus; NORSE = new-onset refractory status epilepticus.*

**SUPPLEMENTARY FIGURE 2**


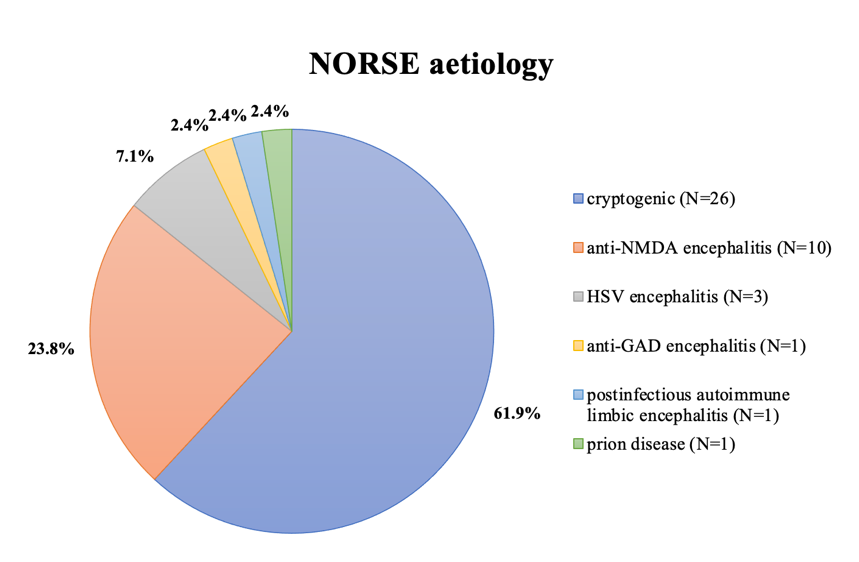


**Supplementary Figure 2:** Graph representing the breakdown of cohort aetiologies, demonstrating the final diagnoses of all NORSE patients. Cryptogenic NORSE (cNORSE) represents 61.9% of the diagnoses, while symptomatic NORSE (sNORSE) was present in the remaining 38.1%, and includes five different diagnostic categories.

*Abbreviations: NMDA = N-methyl-D-aspartate; GAD = glutamic acid decarboxylase; HSV = herpes simplex virus.*

**SUPPLEMENTARY FIGURE 3**

*
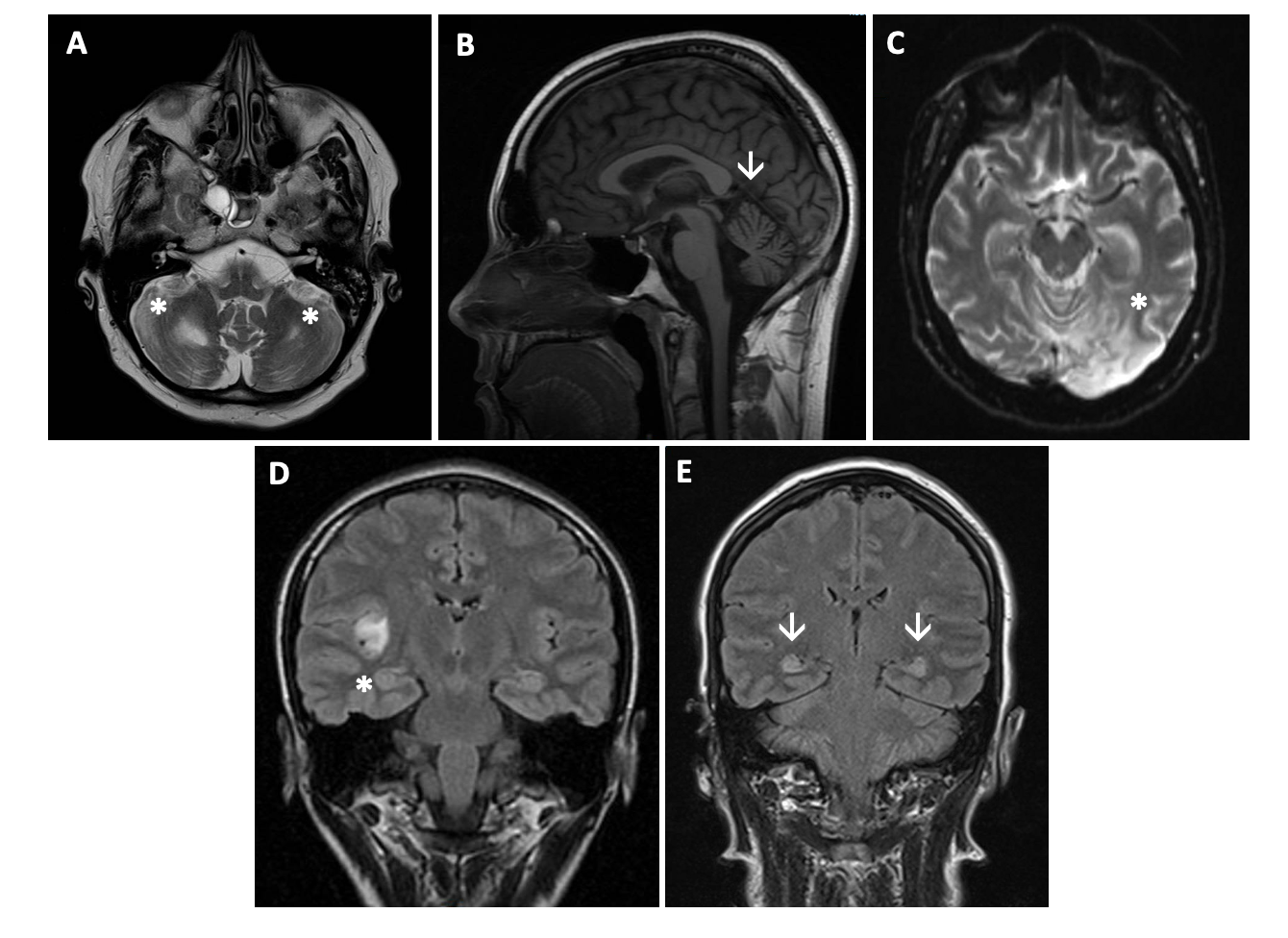
*

**Supplementary Figure 3:** Brain MRI of patients with RSE and primary mitochondrial disease **(A-C)**, sNORSE **(D)**, and cNORSE **(E)**. In **A**, T2 horizontal section showing chronic bilateral metabolic infarcts of cerebellum (white asterisks) in a patient with autosomal recessive *POLG*-related mitochondrial disease. In **B** and **C**, m.3243A>G related mitochondrial disease patient with cerebellar atrophy (T1 sagittal section, white arrow) and acute stroke-like episode in the left occipital lobe (diffusion horizontal section, white asterisk). In **D**, coronal section of a patient with HSV-encephalitis demonstrates abnormal T2 high signal in the insular, claustrum, and adjacent white matter on the right (white asterisk). In **E**, coronal section of a patient with cNORSE showing non-specific bilateral hyperintense hippocampi (white arrows) related to recent seizure activity.

**SUPPLEMENTARY TABLE 1**

|  | **NORSE**  **(N° = 42)** | **cNORSE**  **(N° = 26)** | **sNORSE**  **(N° = 16)** | p-value  (cNORSE vs sNORSE) |
| --- | --- | --- | --- | --- |
| *No. of patients (%) or median (IQR)* | | | | |
| ***Age, y*** | 28 (23-42.5) | 26.5 (23-39.8) | 30 (22.5-44.3) | 0.61 |
| ***Sex (% F)*** | 25 (59.5) | 16 (65.4) | 9 (56.3) | 0.73 |
| ***ICU admission, d*** | 72 (29.5-108.3)^a^ | 60 (25.8-110.8)^b^ | 85.5 (53.8-97.8)^c^ | 0.45 |
| ***Hospital admission, d*** | 105 (58-199.5)^a^ | 91 (49.8-163)^b^ | 172.5 (85.8-243)^c^ | 0.16 |
| ***Number of ASMs used*** | 4 (3-5) | 4 (4-5) | 3 (1.8-4) | **0.01** |
| ***Number of anaesthetics used*** | 3 (2-3) | 3 (2-3) | 2 (2-2.5) | **0.01** |
| ***Mortality during admission*** | 6 (15.4)^d^ | 3 (12.5)^e^ | 3 (20.0)^f^ | 0.53 |
| ***Total mortality 1y post-discharge*** | 8 (20.5)^d^ | 5 (20.8)^e^ | 3 (20.0)^f^ | 0.95 |
| ***Total mortality through July 2022*** | 12 (30.8)^d^ | 9 (37.5)^e^ | 3 (20.0)^f^ | 0.25 |
| ***mRS score, discharge*** | 4 (4-5)^a^ | 4.5 (4-5)^b^ | 4 (4-5)^c^ | 0.91 |
| ***mRS score, 1y*** | 4 (2-5)^g^ | 5 (3-5.5)^h^ | 3 (1.3-4.5)^c^ | 0.10 |
| ***IMD investigations (biochemical or molecular)***  *PMD investigations only* | 21 (50)  10 (23.8) | 19 (73.1)  9 (34.6) | 2 (12.5)  1 (6.3) | **0.00006**  **0.036** |

**Supplementary Table 1:** Comparison of patient demographics, outcomes and investigations in cNORSE and sNORSE patient populations. Significant p-values are in bold.

Number of subjects with available data (if not specified, data is available for all cases): ^a^N=36, ^b^N=22, ^c^N=14, ^d^N=39, ^e^N=24, ^f^N=15, ^g^N=33, ^h^N=19

Abbreviations: NORSE = new-onset refractory status epilepticus; cNORSE = cryptogenic NORSE; sNORSE = symptomatic NORSE; IQR = interquartile range; ICU = intensive care unit; ASM = anti-seizure medication; mRS = modified Rankin Scale; IMD = inherited metabolic disorders; PMD = primary mitochondrial disease; y = year(s); d = days.

**SUPPLEMENTARY TABLE 2**

| *Pt* | *Age (y range)* | | *ICU admission (d)* | *Hospital admission (d)* | | *Prodromal symptoms* | *mRS score (discharge)* | | *mRS score (1y follow-up)* |
| --- | --- | --- | --- | --- | --- | --- | --- | --- | --- |
| *CRYPTOGENIC (cNORSE)* | | | | | | | | | |
| 1* | 20s | 112 | | 172 | headache, sore throat, myalgia, fever, 2 GTCS witnessed | | | 3 | not yet 1 yr |
| 2* | 18-20 | 521 | | 915 | x7 days earache, sore throat, fever, headache, confusion | | | 5 | 5 |
| 3* | 20s | 113 | | 166 | fever, headache, lethargy, non-blanching petechial rash on face | | | 4 | 3 |
| 4* | 18-20 | 28 | | 154 | none | | | 5 | 5 |
| 5* | 20s | 30 | | 48 | increased frequency of visual aura (seeing hexagons) | | | 4 | no f/u |
| 6* | 40s | 99 | | 138 | x2 days headache, confusion | | | 4 | 4 |
| 7* | 20s | 170 | | 220 | flu-like prodrome | | | 5 | 5 |
| 8* | 60s | 53 | | 91 | migraine, left facial and hemibody weakness, confusion, slurred speech, agitation | | | 6 | 6 |
| 9* | 20s | 235 | | 364 | prodrome of non-specific viral illness | | | 5 | 5 |
| 10 | 20s | 7 | | 16 | x2 days fatigue, mild fever | | | 3 | 2 |
| 11 | 50s | 25 | | 55 | GTCS witnessed | | | 4 | 6 |
| 12 | 40s | 18 | | 29 | reported feeling 'hung-over' night before SE, GTCS witnessed | | | 2 | 1 |
| 13 | 18-20 | 21 | | 36 | x1 month headache, several days of probable focal seizures with secondary generalization | | | 5 | 6 |
| 14 | 50s | 107 | | 144 | x2-3 days myalgia, fevers, poor sleep/insomnia, acute confusion | | | 4 | 3 |
| 15 | 40s | 2 | | 10 | short flu-like prodrome | | | 6 | 6 |
| 16 | 30s | 34 | | 61 | x5 days confusion, sore throat, mild speech disturbances | | | 4 | 4 |
| 17 | 30s | 8 | | 36 | x1 day lethargy, numb left arm/face, headache | | | 6 | 6 |
| 18 | 30s | 49 | | 59 | x5 days sore throat, fever, malaise, cervical lymphadenopathy | | | 5 | 2 |
| 19 | 20s | 67 | | 84 | none | | | 5 | 5 |
| 20 | 20s | 72 | | 91 | fever, headache, shaking and frothing at the mouth (self-terminated) | | | 2 | no f/u |
| 21 | 30s | UNK | | UNK | UNK | | | UNK | UNK |
| 22 | 20s | 103 | | 118 | headache, fever, nausea, malaise | | | 3 | no f/u |
| 23 | 20s | 314 | | 384 | x1 month fever, headaches, confusion, forgetfulness | | | 5 | 5 |
| 24 | 20s | UNK | | UNK | UNK | | | UNK | no f/u |
| 25 | 40s | UNK | | UNK | UNK | | | UNK | 2 |
| 26 | 30s | UNK | | UNK | UNK | | | UNK | UNK |
| *SYMPTOMATIC (sNORSE)* | | | | | | | | | |
| *AUTOIMMUNE ENCEPHALITIS* | | | | | | | | | |
| 27* | 20s | 85 | | 92 | witnessed jerking of right leg, version of neck to the left, progressing to foaming with jerks | | 4 | | 1 |
| 28 | 40s | 259 | | 450 | x2 days headache, ash on arms/legs, intermittent fevers | | 4 | | 2 |
| 29 | 20s | 86 | | 162 | unwitnessed seizures, aggression, disorientation | | 4 | | no f/u |
| 30 | 18-20 | 458 | | 463 | x4 weeks of behaviour changes (agitation, confusion) | | 6 | | 6 |
| 31 | 20s | 325 | | 325 | UNK | | 6 | | 6 |
| 32 | 20s | 72 | | 194 | personality changes, viral illness with cough, fever, myalgia, headache | | 5 | | 3 |
| 33 | 20s | 98 | | 252 | reduced appetite, night sweats, dysarthria, dysphasia, GTCS witnessed | | 4 | | 1 |
| 34 | 40s | 35 | | 88 | earache, headache, chest cold symptoms, photophobia, malaise, anorexia, progressed to abnormal behaviour, restlessness, agitation/aggression, sweating and screaming | | 2 | | 1 |
| 35 | 30s | 87 | | 183 | x3 months intermittent headaches, nausea/vomiting, GTCS witnessed | | 5 | | 3 |
| 36 | 40s | UNK | | UNK | UNK | | UNK | | UNK |
| 37 | 20s | 97 | | 216 | x5 days fever, confusion | | 4 | | 1 |
| 38 | 50s | 62 | | 85 | x2 weeks flu-like illness, absence seizure followed by focal and GTCS episodes witnessed | | 4 | | 3 |
| *VIRAL ENCEPHALITIS* | | | | | | | | | |
| 39 | 40s | 14 | | 23 | x2 days malaise, headache, myalgia | | 4 | | 3 |
| 40 | 80s | 51 | | 64 | x24 hours confusion, dysphasia, right-sided facial droop, 2 seizures witnessed | | 5 | | 5 |
| 41 | 20s | UNK | | UNK | UNK | | UNK | | 2 |
| *PRION DISEASE* | | | | | | | | | |
| 42 | 50s | 23 | | 37 | x1 week headache, unsteady gait/shaking, abnormal behaviour, reduced appetite | | 6 | | 6 |

**Supplementary Table 2.** NORSE patient demographics. Table summarising the sex, age at presentation, length of ICU stay and hospital admission, prodromal symptoms experienced, and outcomes at discharge and one-year post-discharge in all 42 NORSE patients. The age at presentation is reported as “year range” to avoid subject identification.

* = Patients with mitochondrial genetics performed.

Abbreviations: f/u = follow-up; ICU = intensive care unit; mRS = modified Rankin Scale; GTCS = generalised tonic-clonic seizure; UNK = unknown.

**SUPPLEMENTARY TABLE 3**

| Pt | N°. ASMs | | ASM type | N°. ana-esthetics | | Anaesthetic type | Immuno-therapy | Immunotherapy type |
| --- | --- | --- | --- | --- | --- | --- | --- | --- |
| *CRYPTOGENIC (cNORSE)* | | | | | | | | |
| 1 | 5 | CBZ, LCM, LEV, PHT, VPA | | 5 | FEN, KET, MDZ, PPF, TP | | Y | IVIG, MP, PLEX, RIT |
| 2 | 4 | LCM, LEV, PHB, PHT | | 5 | FEN, KET, MDZ, PPF, TP | | Y | ANK, MP, PLEX, PSL |
| 3 | 4 | CLB, LEV, PHT, VPA | | 3 | FEN, MDZ, PPF | | N |  |
| 4 | 6 | CLB, LEV, LTG, PER, PHT, TPM | | 0 |  | | Y | PSL |
| 5 | 4 | CBZ, LEV, TPM, VPA | | 3 | FEN, MDZ, PPF | | N |  |
| 6 | 7 | CBZ, LCM, LEV, PER, PHB, PLD, TPM | | 3 | FEN, MDZ, PPF | | Y | IVIG, MP, PLEX |
| 7 | 6 | CLB, LCM, LEV, PER, PHB, PHT | | 3 | FEN, MDZ, TP | | Y | IVIG, MP |
| 8 | 4 | CLB, LCM, LEV, PHT | | 3 | FEN, MDZ, PPF | | Y | IVIG, MP, PLEX, PSL |
| 9 | 6 | LCM, LEV, LTG, PHB, PHT, VPA | | 2 | MDZ, TP | | Y | MP, PSL |
| 10 | 4 | CBZ, CLB, LEV, LZP | | 2 | FEN, PPF | | N |  |
| 11 | 4 | LCM, LEV, PHT, VPA | | 2 | FEN, PPF | | N |  |
| 12 | 5 | DZP, LEV, LZP, PHT, VPA | | 3 | FEN, MDZ, PPF | | N |  |
| 13 | 3 | LCM, LEV, PHT | | 3 | FEN, MDZ, PPF | | N |  |
| 14 | 5 | CLB, LCM, LEV, PER, PHT | | 3 | FEN, MDZ, PPF | | Y | MP, PSL |
| 15 | 3 | CBZ, LEV, PHT | | 3 | MDZ, PPF, TP | | N |  |
| 16 | 5 | LEV, PHB, PHT, TPM, VPA | | 3 | ALF, MDZ, PPF | | Y | IVIG, PLEX |
| 17 | 4 | LCM, LEV, PHB, PHT | | 2 | MDZ, TP | | Y | IVIG, MP |
| 18 | 7 | CLB, LEV, LCM, PER, PHB, PHT, TPM | | 3 | FEN, MDZ, PPF | | Y | PSL |
| 19 | 3 | PHT, TPM, VPA | | 2 | REMI, PPF | | N |  |
| 20 | 4 | LEV, PHB, PHT, TPM | | 4 | FEN, MDZ, PPF, TP | | N |  |
| 21 | 3 | CLN, PHB, PHT | | NA |  | | N |  |
| 22 | 3 | LEV, PHB, PHT | | 5 | FEN, KET, MDZ, PPF, TP | | Y | MP, PSL |
| 23 | 4 | CBZ, CLB, LEV, PHT | | 1 | PPF | | N |  |
| 24 | 4 | LEV, LTG, MgSO4, TPM | | 4 | KET, MDZ, PPF, TP | | Y | IVIG, MP, PSL |
| 25 | 2 | PHT, VPA | | 3 | FEN, MDZ, PPF | | N |  |
| 26 | 4 | LEV, PHB, PHT, TPM | | NA |  | | Y | MP, PSL |
| *SYMPTOMATIC (sNORSE)* | | | | | | | | |
| *AUTOIMMUNE ENCEPHALITIS* | | | | | | | | |
| 27 | 5 | LEV, PER, PHB, PHT, VPA | | 3 | FEN, MDZ, PPF | | Y | IVIG, MP, PLEX, PSL, RIT |
| 28 | 3 | CLB, LEV, PHT | | 3 | FEN, MDZ, PPF | | Y | BTZ, IVIG, MP, PLEX, RIT |
| 29 | 4 | DZP, LEV, PHT, VPA | | 2 | FEN, PPF | | Y | CPM |
| 30 | 5 | LCM, LEV, PHB, PHT, VPA | | 2 | FEN, PPF | | Y | PLEX |
| 31 | 2 | CLN, LEV | | 1 | PPF | | Y | PLEX |
| 32 | 1 | PHT | | 2 | MDZ, PPF | | Y | IVIG |
| 33 | 3 | LEV, PHB, PHT | | 2 | FEN, MDZ | | N |  |
| 34 | 1 | PHT | | 0 |  | | Y | IVIG, MP, PSL |
| 35 | 1 | CLN | | 2 | MDZ, PPF | | Y | IVIG, MP, PLEX, RIT |
| 36 | 4 | CLN, LEV, PHB, PHT | | NA |  | | Y | IVIG, MP, PLEX, PSL |
| 37 | 4 | LEV, PHT, TPM, VPA | | 2 | FEN, PPF | | Y | PLEX |
| 38 | 5 | CLN, LCM, LEV, PHT, VPA | | 3 | FEN, MDZ, PPF | | Y | IVIG, MP, PLEX, PSL |
| *VIRAL ENCEPHALITIS* | | | | | | | | |
| 39 | 3 | CLB, LEV, PHT | | 3 | FEN, PPF, TP | | N |  |
| 40 | 1 | LEV | | 2 | FEN, PPF | | N |  |
| 41 | 3 | LEV, PHT, VPA | | 0 |  | | N |  |
| *PRION DISEASE* | | | | | | | | |
| 42 | 3 | LEV, PHT, VPA | | 2 | MDZ, PPF | | N |  |

**Supplementary Table 3.** Table summarising individual NORSE patient treatments, including number and type of ASMs, anaesthetics and immunotherapies used to treat status epilepticus.

Abbreviations: ALF = alfentanil; ANK = anakinra; ASM = anti-seizure medication; BRV = brivaracetam; BTZ = bortezomib; CBZ = carbamazepine; CLB = clobazam; CLN = clonazepam; CPM = cyclophosphamide; DZP = diazepam; FEN = fentanyl; IVIG = intravenous immunoglobulins; MP = methylprednisolone; KET = ketamine; LCM = lacosamide; LEV = levetiracetam; LTG = lamotrigine; LZP = lorazepam; MDZ = midazolam; MgSO4 = magnesium sulfate; NA = not available; PER = perampanel; PHB = phenobarbital; PHT = phenytoin; PLD = paraldehyde; PLEX = plasma exchange; PPF = propofol; PSL = prednisolone; REMI = remifentanil; RIT = rituximab; TP = thiopental; TPM = topiramate; VPA = valproate.

**SUPPLEMENTARY TABLE 4**

| Pt | CSE or NCSE? | EEG | Brain MRI | MRI comments | CSF protein (0.13-0.45 g/L) | CSF WCC (0-5) | CSF RCC (<1) | CSF oligo-clonal bands | CSF lactate (0.6-3.1 mmol/L) | CSF viral PCR (+/-) | CSF bacterial cultures (+/-) | CSF neurodegenerative biomarkers: total tau (146-595 pg/mL), 14.3.3 protein |
| --- | --- | --- | --- | --- | --- | --- | --- | --- | --- | --- | --- | --- |
| *CRYPTOGENIC (cNORSE)* | | | | | | | | | | | | |
| 1 | NCSE |  |  | Symmetrical hyperintensities in bilateral hippocampi and the posteromedial thalamic regions. |  |  | 1 | (-) | UNK | (-) | (-) |  |
| 2 | CSE |  |  | Non-specific diffuse leptomeningeal enhancement. Hyperintensities in bilateral hippocampi and left amygdala. | 0.53 |  |  | (-) | 5.34 (H) | (-) | (-) | total tau = 10955. 14.3.3 positive. |
| 3 | CSE |  |  |  |  |  | 7 | UNK | 210 (H) | (-) | (-) |  |
| 4 | NCSE |  |  | Known neurodegenerative condition, unknown aetiology. Progression of volume loss and cortical signal change, new signal change in the right frontal operculum. |  |  |  | (+) |  | (-) | (-) | total tau = 638. 14.3.3 negative. |
| 5 | UNK |  |  | Symmetrical hyperintensities in the cortices of the bilateral hippocampi, anterior temporal poles and insula. |  |  |  | (-) |  | (-) | (-) |  |
| 6 | UNK |  |  |  | 1.82 |  |  | (-) |  | (-) | (-) |  |
| 7 | UNK |  |  | Patchy temporal lobe enhancement, hyperintensity in thalamus with some substantia nigra involvement. |  |  |  | (-) |  | (-) | (-) |  |
| 8 | CSE |  |  | Symmetrical cerebral volume loss, especially in bilateral hippocampi. |  |  |  | (+) | UNK | (-) | (-) | total tau = 649. 14.3.3 positive. |
| 9 | CSE |  |  |  |  |  | UNK | (-) | UNK | (-) | UNK |  |
| 10 | CSE |  |  |  |  |  | UNK | UNK |  | (-) | (-) |  |
| 11 | CSE |  |  |  | 1.09 |  | <1 | (+) |  | (-) | (-) |  |
| 12 | UNK |  |  |  |  |  | 24 | UNK | UNK | (-) | (-) |  |
| 13 | UNK |  |  | Hyperintensity of bilateral hippocampi. |  | 8 | <1 | (-) |  | (-) | (-) |  |
| 14 | NCSE |  |  |  | 0.5 |  | 7 | UNK |  | (-) | (-) |  |
| 15 | UNK |  |  | Hyperintensity and possible enhancement of right middle temporal gyrus. |  |  | 32 | UNK | UNK | (-) | moderate growth of staphylococcus |  |
| 16 | CSE |  |  |  | 0.47 |  | 1 | UNK | UNK | (-) | (-) |  |
| 17 | UNK |  |  |  |  |  | UNK | UNK | UNK | (-) | (-) |  |
| 18 | CSE |  |  | Hyperintensity of bilateral hippocampi. |  |  | UNK | (-) |  | (-) | (-) |  |
| 19 | UNK |  |  |  |  |  | 1 | UNK |  | (-) | (-) |  |
| 20 | NCSE |  |  |  |  | 16 |  | UNK | UNK | (-) | (-) |  |
| 21 | UNK |  |  | Signal change in right temporal lobe. | UNK | UNK | UNK | UNK |  | UNK | UNK |  |
| 22 | CSE |  |  |  |  | 10 | UNK | UNK |  | (-) | (-) |  |
| 23 | CSE |  |  |  |  | 8 | UNK | UNK | UNK | (-) | (-) |  |
| 24 | NCSE |  |  |  |  | 11 | UNK | UNK |  | UNK | UNK |  |
| 25 | UNK |  |  | Hyperintensity of bilateral hippocampi. |  | 14 | UNK | UNK |  | UNK | UNK |  |
| 26 | UNK |  |  | Hyperintensity throughout the cerebellum, hippocampi, medial right temporal lobe. Leptomeningeal enhancement. Non-specific hyperintensities in subcortical and deep white matter in both frontal lobes. | UNK | UNK | UNK | UNK | UNK | UNK | UNK |  |
| *SYMPTOMATIC (sNORSE)* | | | | | | | | | | | | |
| *AUTOIMMUNE ENCEPHALITIS* | | | | | | | | | | | | |
| 27 | CSE |  |  | Abnormal signal changes in the left insula, Herschel’s lobe and temporal pole; multifocal parenchymal lesions involving the left hippocampus, amygdala and left superior frontal gyrus. Unequivocal rapid progression of the imaging findings when compared to the previous MRI performed at presenting hospital 13 days before. |  |  |  | (+) |  | (-) | moderate growth of E.coli |  |
| 28 | NCSE |  |  | Signal changes in mesial temporal lobe and right parahippocampal gyrus enhancement. | 1.17 | 120 | 35 | (+) | UNK | (-) | (-) |  |
| 29 | UNK |  |  | NA | UNK | UNK | UNK | UNK | UNK | (-) | (-) |  |
| 30 | NCSE |  |  | Possible temporal lobe enhancement, subtle loss of neuroparenchymal volume. |  | 35 | 4 | UNK | UNK | (-) | (-) |  |
| 31 | UNK |  |  |  |  | 10 | 58 | UNK | UNK | (-) | (-) |  |
| 32 | UNK |  |  | Normal intracranial appearances on admission. Imaging 2 weeks later demonstrated multifocal signal abnormalities in the bilateral parahippocampal gyri, olfactory gyri, right lateral orbital lobule and splenium of the corpus callosum and diffuse leptomeningeal enhancement. |  | 29 | 2250 | (+) |  | (-) | (-) | Total tau >1200. 14.3.3 positive. |
| 33 | UNK |  |  |  |  | 57 | UNK | UNK | UNK | (-) | (-) |  |
| 34 | UNK |  |  | Persistent lack of CSF suppression on the FLAIR sequences. Linear enhancement of the dural meninges. |  | 13 | 2 | (+) | UNK | (-) | (-) |  |
| 35 | UNK |  |  |  | 0.55 | 12 | 1 | (+) |  | UNK | UNK |  |
| 36 | UNK |  |  |  | UNK | UNK | UNK | UNK |  | UNK | UNK |  |
| 37 | NCSE |  |  |  |  | 80 | UNK | (-) | UNK | (-) | (-) |  |
| 38 | UNK |  |  | Hyperintensity of bilateral hippocampi. |  |  | UNK | UNK |  | (-) | (-) |  |
| *VIRAL ENCEPHALITIS* | | | | | | | | | | | | |
| 39 | NCSE |  |  | Bilateral signal changes in medial temporal and medial frontal gyrus with gyriform restricted diffusion; later showed enhancement within some of these regions. | 1.03 | 41 | UNK | (+) | UNK | (+) HSV-1 | (-) |  |
| 40 | NCSE |  |  | Cortical swelling and associated restricted diffusion in left insular cortex, frontal operculum, sub-central gyrus, left cingulate, superior left parietal lobe and right insular cortex. |  |  |  | (+) | UNK | (+) HSV-1 | (-) |  |
| 41 | NCSE |  |  | Signal changes in the insular, claustrum and adjacent white matter extending down to the temporal stem on the right side. | UNK | 12 | 800 | UNK | UNK | (+) HSV-1 | (-) |  |
| *PRION DISEASE* | | | | | | | | | | | | |
| 42 | UNK |  |  | Signal changes and restricted diffusion in the caudate and putamen. | 0.59 |  |  | UNK | UNK | (-) | UNK |  |

**Supplementary Table 4.** Results of NORSE patient EEG, MRI and CSF testing on admission. Abnormal results are indicated in red, normal results are indicated in green. Yellow indicates an abnormal MRI result attributed to seizure activity rather than causative structural damage, according with expert neuroradiologists. Testing not completed or with no accessible results labelled unknown (UNK) or left blank (last column).

Abbreviations: CSE = convulsive status epilepticus; HSV-1 = herpes simplex virus type 1; NCSE = non-convulsive status epilepticus; PCR = polymerase chain reaction; RCC = red cell count; WCC = white cell count.

**SUPPLEMENTARY FIGURE 4**


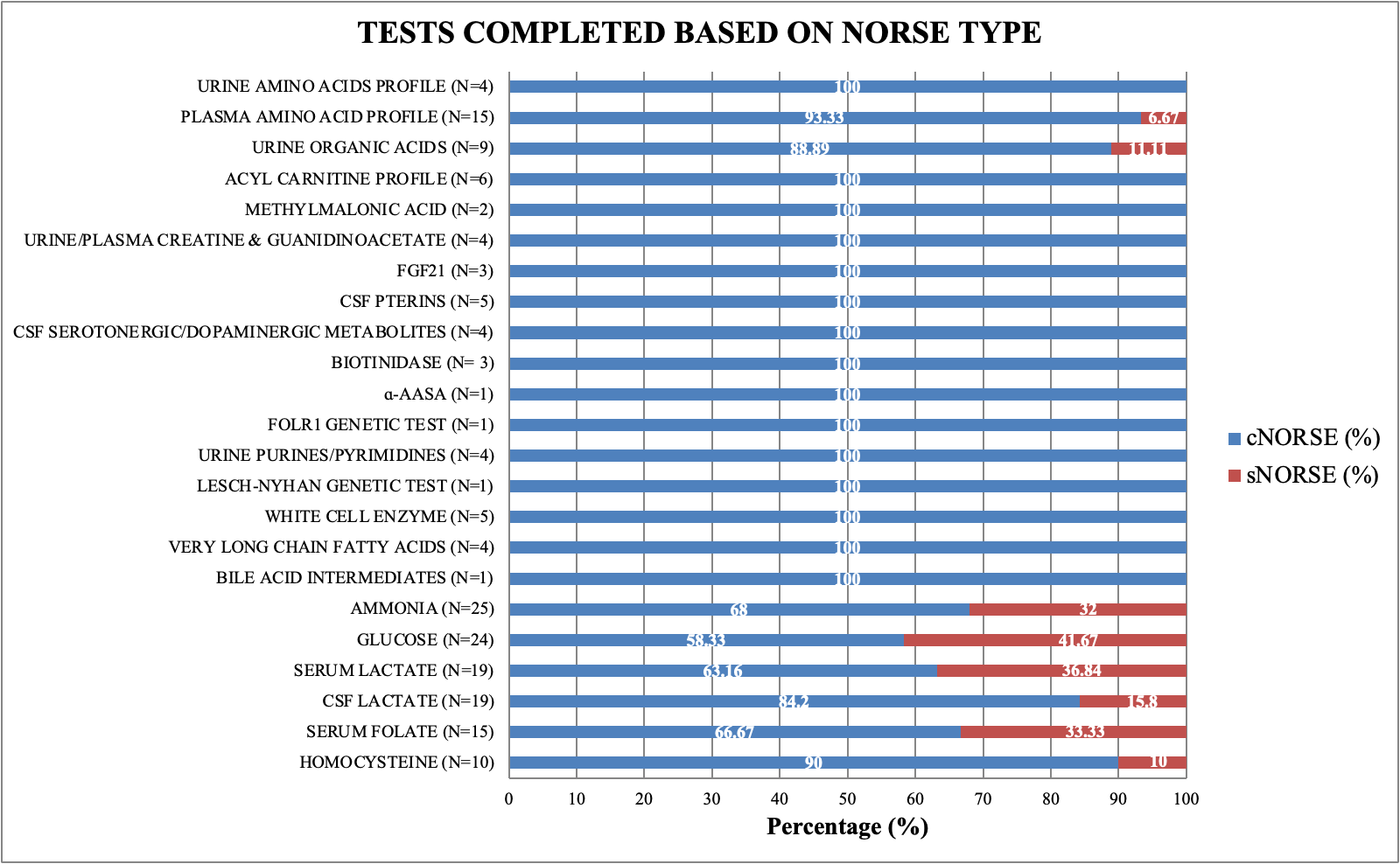


**Supplementary Figure 4.** Metabolic tests (IMD second-line investigations and routine standard metabolic tests) completed based on NORSE type. See also Supplementary Table 5 for mitochondrial genetic test results.

*Abbreviations: ɑ-AASA = Alpha-aminoadipic semialdehyde dehydrogenase; FGF21 = fibroblast growth factor 21; FOLR1 = folate receptor alpha gene.*

**SUPPLEMENTARY TABLE 5**

| Pt | EMG result | EMG - comment | Muscle biopsy result | Muscle biopsy - comment | RCEA result | Mitochondrial genetic testing result | Sample source | Mitochondrial genetic testing - comment | Other genetic tests (metabolic / non-metabolic) |
| --- | --- | --- | --- | --- | --- | --- | --- | --- | --- |
| *CRYPTOGENIC (cNORSE)* | | | | | | | | | |
| 1 |  |  |  |  |  |  | Blood, urine | Common mtDNA mutations, mtDNA sequencing, maintenance panel | Targeted karyotyping, ring chromosome 20 |
| 2 |  |  |  | Disuse atrophy |  |  | Blood, urine, muscle | Common mtDNA mutations, mtDNA sequencing, large  scale mtDNA rearrangements,  mtDNA depletion,  maintenance panel | Factor V Leiden mutation,  Factor II prothrombin mutation |
| 3 |  | CINM |  |  |  |  | Blood | Common mtDNA mutations, WES |  |
| 4 |  |  |  |  |  |  | Blood, muscle | Common mtDNA mutations, WES | *FOLR1*, epilepsy panel |
| 5 |  |  |  |  |  |  | Muscle | Common mtDNA mutations, large scale mtDNA rearrangements | *PCDH19* |
| 6 |  | CINM |  |  |  |  | Blood | Common mtDNA mutations, maintenance panel | Factor II prothrombin, Factor V Leiden |
| 7 |  | CINM |  |  |  |  | Muscle | Common mtDNA mutations, large  scale mtDNA rearrangements, maintenance panel | Ring chromosome 20 |
| 8 |  |  |  |  |  |  | Muscle | Common mtDNA mutations | Prion protein genetic analysis |
| 9 |  | CINM |  |  |  |  | Blood, muscle | Common mtDNA mutations, maintenance panel | Lesch-Nyhan (*HPRT*), *SCN1A*, Rett syndrome (*MECP2*), ring chromosome 20 |
| 14 |  | CIM |  |  |  |  |  |  |  |
| 16 |  | CIN |  |  |  |  |  |  |  |
| 19 |  |  |  |  |  |  |  |  |  |
| 22 |  | CIN |  |  |  |  |  |  |  |
| *SYMPTOMATIC (sNORSE)* | | | | | | | | | |
| *AUTOIMMUNE ENCEPHALITIS* | | | | | | | | | |
| 27 |  | CIM |  |  |  |  | Blood | Common mtDNA mutations |  |
| 28 |  | CIM |  |  |  |  |  |  |  |
| 33 |  | CINM |  |  |  |  |  |  |  |
| 34 |  | CINM |  |  |  |  |  |  |  |

**Supplementary Table 5.** Results of EMG, muscle biopsy, RCEA, and genetic testing. Abnormal results are indicated in red, normal results are indicated in green. Testing not completed left blank. The absence of a patient number indicates that none of the specified tests were conducted for that individual.

Abbreviations: CIM = critical illness myopathy; CIN = critical illness neuropathy; CINM = critical illness neuromyopathy; mtDNA = mitochondrial DNA; RCEA = respiratory chain enzyme analysis; WES = whole exome sequencing. Name of genes are italicised.
